# Lessons from inducible pluripotent stem cell models on neuronal senescence in aging and neurodegeneration

**DOI:** 10.1038/s43587-024-00586-3

**Published:** 2024-03-01

**Authors:** Isabelle de Luzy, Micheal Lee, William Mobley, Lorenz Studer

**Affiliations:** 1 The Center for Stem Cell Biology and Developmental Biology Program, Sloan-Kettering Institute for Cancer Research, 1275 York Avenue, New York, NY, 10065, USA.; 2 Department of Neuroscience, University of Minnesota, 4-125 Jackson Hall, 321 Church Street SE, Minneapolis, MN, 55455, USA.; 3 Institute for Translational Neuroscience, University of Minnesota, 2101 6th Street SE, Minneapolis, MN, 55455, USA.; 4 Department of Neurosciences, University of California San Diego, La Jolla, California, USA.; 5 Aligning Science Across Parkinson’s (ASAP) Collaborative Research Network, Chevy Chase, MD, 20815, USA.

## Abstract

Age remains the central risk factor for many neurodegenerative diseases including Parkinson’s disease, Alzheimer’s disease and Amyotrophic lateral sclerosis. While the mechanisms of aging are complex, the age-related accumulation of senescent cells in neurodegeneration is well documented and their clearance can alleviate disease-related features in preclinical models. Senescence-like characteristics are observed in both neuronal and glial lineages, but their relative contribution to aging and neurodegeneration remains unclear. Human pluripotent stem cell (hPSC)-derived neurons provide an experimental model system to induce neuronal senescence. However, the extensive heterogeneity in the profile of senescent neurons and the methods to assess senescence remain major challenges. Here, we will review the evidence of cellular senescence in neuronal aging and disease, discuss hPSC-based model systems used to investigate neuronal senescence and propose a panel of cellular and molecular hallmarks to characterise senescent neurons. Understanding the role of neuronal senescence may yield novel therapeutic opportunities in neurodegenerative disease.

## Introduction

Several cellular and molecular hallmarks of aging have been described^1^, many of which may apply to neurons and other CNS cell types. These include genomic instability, telomere attrition, epigenetic alterations, loss of proteostasis, disabled macroautophagy, dysregulated nutrient sensing, mitochondrial dysfunction, stem cell exhaustion, altered intercellular communication, chronic inflammation, dysbiosis and cellular senescence. Which of those hallmarks are drivers rather than markers of age remains an important question in the field.

**Figure 1.**
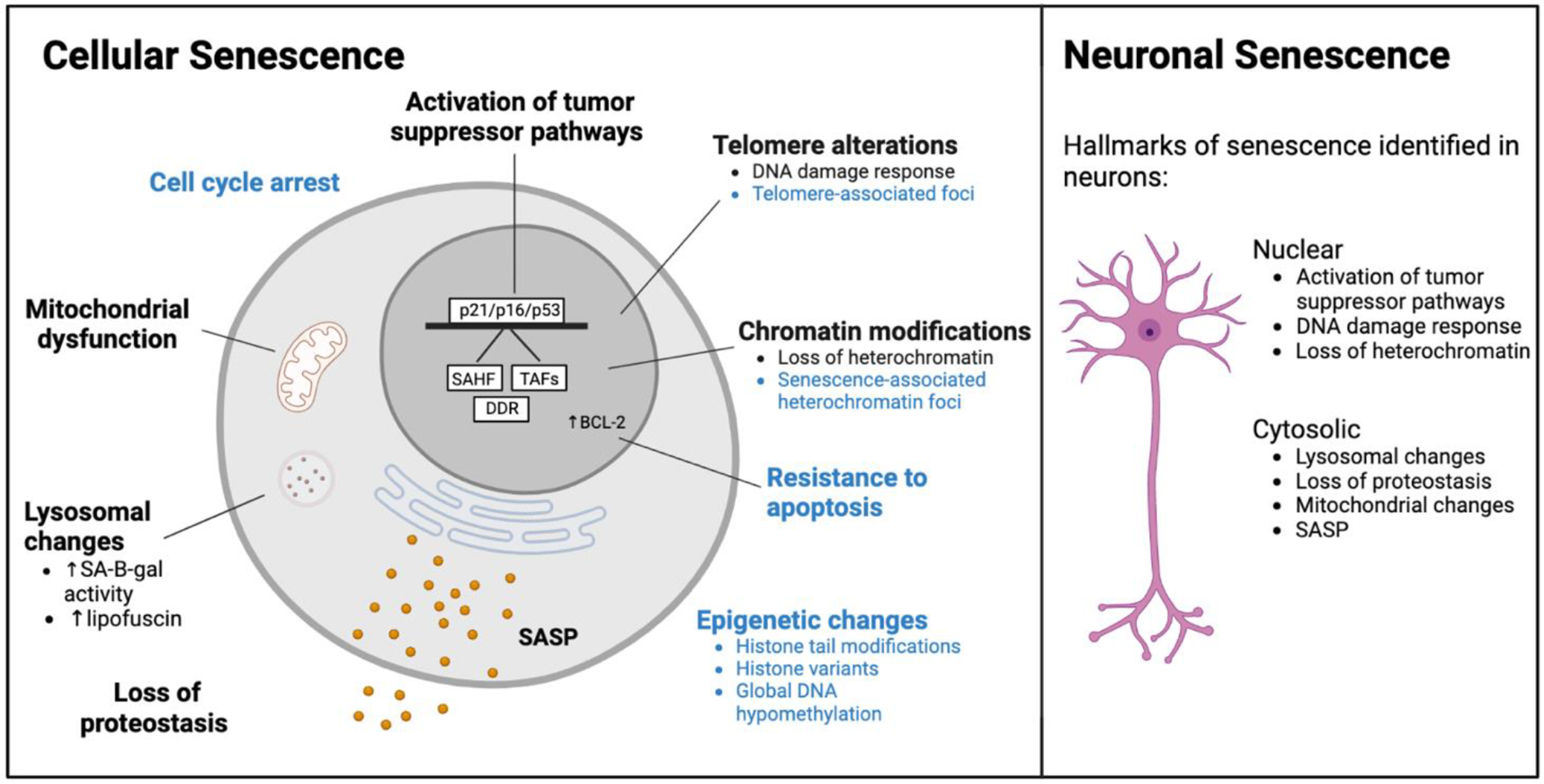
Established hallmarks of cellular senescence and those commonly identified in aged neurons and neurodegenerative neurons. Among the cellular senescence phenotypes presented in the schematic, those that have been conclusively detected in neurons are marked in black.

The term senescence was first coined in the context of replicative senescence more than 60 years ago by Leonard Hayflick and Paul Moorhead. This landmark study revealed that human fibroblasts have a finite capacity for cellular replication *in vitro*^2^ due to progressive telomere erosion^3^. Over the following years, studies have revealed cellular senescence as a more complex biological process that is multifactorial and dynamic. Cellular senescence is characterised by a permanent state of cell cycle arrest and the secretion of a plethora of pro-inflammatory secretory molecules, known as a senescence-associated secretary phenotype (SASP)^4^. The SASP secretome is composed of cytokines including chemokines, proteases and growth factors^5^. Interestingly, there is considerable overlap in the secretory profile of senescent cells with that of activated immune cells in the CNS^6,7^, suggesting an interaction between senescence and neuroinflammation. Importantly, the secretion of SASP factors can trigger senescence in neighbouring healthy cells^6,8^, which may be a driver of aging and age-related pathologies. Other common cellular and molecular features of senescence include activation of tumour suppressor pathways (p16/retinoblastoma, p53/p21), resistance to apoptosis, changes in morphology and lysosomal activity, as well as a myriad of epigenetic alterations (Figure 1)^5,9^. Whilst cellular senescence plays an essential biological role to protect cells against toxic stress; for example, during embryogenesis, following cell transformation in cancer or in response to tissue damage, a persistent senescence signature can have deleterious effects in the body and has been linked to inflammation, aging and disease^10^.

While cellular senescence is considered one of the hallmarks of aging^1^, many of the other hallmarks of aging are also shared with those of cellular senescence (Figure 1) suggesting that those processes are closely connected. For example, several pathways associated with mitochondrial dysfunction have been identified in aging and senescence, as reviewed by Miwa et al^11^. However, there are also several distinctions between cellular senescence and aging raising important questions such as, what is the specific relationship of aging with senescence and vice versa and how are those two features interlinked. For example, senescent cells can arise during embryonic development^12,13^, while many cells in the aged brain are not senescent but still express certain age-related hallmarks, this suggests that senescence and aging can occur independently in some cases. Furthermore, cells that are aging often gradually lose proliferative function while cells that become senescent typically arrest from cell cycle permanently.

The accumulation of senescent cells with age and disease has been documented in many tissues, including the brain^8^. An early link between cellular senescence and aging in the central nervous system (CNS) was suggested by the detection and build-up of lipofuscin pigments in the aging rodent brain^14^. Lipofuscin is an intracellular pigment produced by oxidative alterations of macromolecules, including lysosomal products^15^. It is particularly associated with senescence in post-mitotic cells, such as neurons. Recent reports have determined that numerous cell types in the aging brain including neurons, astrocytes, microglia, oligodendrocyte progenitor cells, ependymal cells, smooth muscle cells and endothelial cells can exhibit molecular and transcriptomic signatures of senescence^16–18^. The involvement of senescent cells in neurodegenerative diseases has gained strong interest from findings that the clearance of these cells in mouse models of neurodegeneration can modulate aspects of disease-associated pathologies^19–23^, suggesting that cellular senescence may contribute to neurodegenerative dysfunction. Therapeutic efforts are now being pursued to assess the potential benefit of clearing senescence cells in AD patients using senolytics (SToMP-AD clinical trial)^24^. However, the cell types in the CNS that directly contribute to aging and neurodegeneration, which of the various cell types becomes senescent first, as well as the mechanisms by which they may influence disease progression are not well understood.

Several reports have demonstrated that post-mitotic neurons can acquire senescent characteristics in aging and neurodegenerative states. In this review, we will discuss the evidence supporting neuronal senescence and a potential pathogenic link to neurodegenerative disease. We will focus particularly on modelling neuronal senescence in pluripotent stem cell models and on defining a panel of cellular and molecular hallmarks for the reliable identification of senescent neurons.

## Neuronal Senescence

### Detection of senescence phenotypes in neurons

A key challenge studying neuronal senescence is the identification of cellular and molecular hallmarks that accurately detect senescence. The term senescence is not easily defined, as there is no single gene that reliably captures the senescent phenotype. As a result, there are numerous markers and signalling pathways associated with senescence which need to be analysed in combination to identify senescent cells *in vitro* and *in vivo*. To add to this, senescent features within and across cell types are inherently heterogeneous and different neuronal or glial cell types do not necessarily share the same senescence profile. Some biomarkers of cellular senescence are also expressed in cell types with an inflammatory or stress response independent of senescence. Collectively, this makes it difficult to determine if senescent mechanisms are shared across cell types, disease state and/or method to induce senescence. Current approaches to measure neuronal senescence therefore entail a plethora of assays to be conducted to identify senescent cells which makes this process highly involved and time consuming (See Table 1). A subset of phenotypes commonly associated with cellular senescence have been reported in both aged neurons and neurons in neurodegenerative disorders including; activation of tumor suppressor pathways, DNA damage response, loss of heterochromatin, loss of proteostasis, mitochondrial dysfunction, lysosomal abnormalities and SASP (Figure 1, black).

While the accurate identification of senescent cells remains a challenge, the development of eigengene networks and modules has enabled more reliable detection of senescent cells in transcriptomic datasets^25^. Eigengene networks describe the relationship between modules that contain groups of interlinked genes^26^. These modules are defined as eigengenes^26^ and are especially valuable in datasets with a low signal to noise ratio. Using this approach, senescent cells can be detected by the expression of genes across eigengene modules, where each module contains a unique collection of genes associated with senescence^25^. However, the composition of modules are highly variable across studies making interpretations and comparisons challenging. To create more consistency in the detection of senescence cells in transcriptomic datasets, a universal senescent module (SenMayo) consisting of 125 senescence genes, has recently been developed from existing datasets of aged tissues, including the mouse brain^17^. The authors demonstrate the responsiveness of the SenMayo gene set in detecting senescence cells across multiple tissues/species and after senescent cell clearance. Of interest, the pre-frontal cortex and dorsal hippocampal brain regions and microglia were identified to be significantly enriched for senescence/SASP genes in aged mouse brain tissue using the SenMayo module. Therefore, it is currently unclear if SenMayo is predictive or suitable to identify neuronal senescence. Another recent study has identified the activation of Senescence Gene (SnG) networks in multiple cell types, including neurons^16^. Whilst these approaches can identify putative senescent neurons, additional orthogonal validation studies should be performed to support the findings.

### Evidence of neuronal senescence

Following the identification of lipofuscin pigments in the aging rodent brain^14^, it was not until much later that the direct co-localisation of senescence markers in neurons was documented. These studies identified neuronal senescence in cultured primary CA3 hippocampal^36^ and cerebellar granule neurons from embryonic rodents^33^, as well as in the hippocampus of aged rodent brains^36^. However, it should be noted that these follow-up studies heavily relied on histochemical detection of SA-β-galactosidase (SA-β-gal) activity, which has been criticised as an unreliable biomarker for neuronal senescence when used in isolation. It is well known that enhanced expression of SA-β-gal can occur during early stages of neuronal development^39^ and in some brain regions of young mice^40^. This suggests that changes in lysosomal activity based on SA-β-gal expression can arise in the absence of bona fide cellular senescence. Nonetheless, subsequent studies have been able to expand on these findings, demonstrating elevation of SA-β-gal activity, in addition to other senescence phenotypes, including increased lipofuscin, oxidative stress, γH2AX (indicative of a DNA damage response), p21 and two SASP factors; IL-6 and MCP-1, in the cortex of aged mice and after prolonged culture of primary rodent cortical neurons^30,31,34^. Importantly, in one of these studies, Moreno-Blas et al confirmed that both neurons and astrocytes from embryonic rodent cortex cultured *in vitro* display features of cellular senescence, indicated by co-labelling of TUJ1 and GFAP with key senescent markers; p21, yH2AX, LaminA/C^31^. This establishment of neuronal senescence is in part attributed to disturbances in autophagy^31^, as has been described in primary fibroblasts^41^.

Progeroid murine models are a valuable tool to investigate the basic mechanisms of aging including neuronal senescence. A commonly used model to study senescence in the brain is the Ercc1^−/Δ^ progeroid mouse model, which accumulates endogenous DNA damage due to a deficiency in the Ercc1-XPF DNA repair endonuclease complex^42^. The accumulation of DNA damage in Ercc1^−/Δ^ mice promotes cellular senescence to arise at a much faster rate, by 4 months of age they show equivalent accumulation of senescent cells to naturally aged 2.5-year old mice^29,42^. Ercc1^−/Δ^ mice have an average lifespan up to 7 months^43^. In terms of neuronal aging, Ercc1^−/Δ^ mice have proportionally smaller brain and spinal cord tissue as a consequence of their reduced body size at birth to those from wild-type (WT) mice^43,44^, as well as an elevation of the senescence markers, p16INK4A and SA-β-gal, in brain tissue^29^. Interestingly, in-depth analysis of the spinal cord of Ercc1 deficient mice, shows degeneration of motor neurons, along with age-dependent motor abnormalities, astrocytosis and microgliosis^43^. In addition, targeted deletion of Ercc1 in nigral dopaminergic neurons in mice results in the degeneration of striatal processes and loss of midbrain dopaminergic (mDA) neurons^45^. Whether degeneration of spinal motor or dopaminergic neurons in this mouse model is a direct result of neuronal senescence or a consequence of the defective DNA damage response still needs to be investigated. These findings indicate that deficiency in DNA repair due to a partial loss of Ercc1 functionality can cause phenotypes of cellular senescence and accelerate biological age but can also alter embryonic development, suggesting that Ercc1 may play an essential role in both aging and development.

An alternative human progeroid syndrome that has been associated with aging and senescence is Hutchinson-Gilford progeria syndrome (HGPS). HGPS is a rare genetic condition that leads to the expression of progerin, a truncated, toxic form of Lamin A. HGPS patients have an average lifespan of less than 20 years^46^ and suffer from a broad range of age-related symptoms in several organ systems. However, they do not display any obvious defects in their cognitive abilities, and the brain appears largely spared from any pathology, a finding that is likely explained by the very low levels of Lamin A expression in the CNS. However, mouse models involving forced expression of progerin in the CNS have suggested that progerin may be associated with neuronal senescence. For example, in the study by Machiela et al., forced expression of progerin in a mouse model of Huntington’s disease (HD) triggered expression of the senescence marker p16^INK4A^ in neurons^47^. However, another study reported that progerin over-expression in hippocampal and cortical neurons resulted in changes in nuclear morphology but no obvious other age-related phenotypes^48^.

Studies of post-mortem tissue have suggested that human neurons can become senescent in aging and neurodegenerative disease states, but accurate detection of senescent cells has been difficult to date. More recently, Dehkordi et al., utilised a novel transcriptomic eigengene approach, discussed previously, to more precisely profile both the proportion and identity of senescent cells in the brain of AD patients based on three independent eigengene modules defined as i) cell cycle arrest, ii) stress response and iii) inflammatory response^25^. Interestingly, the authors identified that the majority (>97%) of senescent cells in the pre-frontal cortex, across two independent transcriptomic datasets are excitatory neurons, whilst other cell types such as astrocytes, endothelial cells and pericytes display an inflammatory profile independent of senescence. It is worth noting that this finding has been replicated in a parallel study focused on the same brain region in sporadic and familial AD patients^6^. Interestingly, Dekhordi et al reported that most of the identified senescent neurons contained tau neurofibrillary tangles, a classical hallmark of AD pathology^25^. This finding is based on previous work by the same group in AD transgenic models showing a close association of tau pathology and neuronal senescence^20^. Interestingly, much earlier studies reported that terminally differentiated neurons in AD brains containing neurofibrillary tangles, show abnormal enhanced expression of the cell cycle regulator p16^49,50^. These studies present a first of a kind in-human effort and may enable the assessment of other brain regions and/or disease contexts using similar approaches to expand our understanding of the senescent cell types involved in neurodegeneration.

Of interest, another study examining senescence features in a tau-dependent mouse model of neurodegeneration found that astrocytes and microglia show evidence of cellular senescence^19^. A caveat in this study is the identification of senescent cells using a p16^INK4A^-reporter despite other reports suggesting that tau-dependent neuronal senescence in the human brain is most associated with an increase in p19^INK4D^ expression^25^. We speculate that the difference in senescent cell types identified between these studies may reflect species heterogeneity, disease severity, the methodology to detect senescence cells and the diversity of senescence markers expressed by senescence cells. For example, a recent report has shown that p21- and p16-expressing senescent cells are found in distinct cell populations across aged tissues^51^ or alternatively there may be a temporal component to senescence with p21 initiated prior to other cell cycle-dependent genes such as CDKN2A or CDKN2D. These discrepancies highlight the importance of developing consistent and rigorous quantitative methods to measure senescence within the brain that enable the field to distinguish between technical variation in readouts versus biological differences.

## In vitro models of human neurons

**Figure 2:**
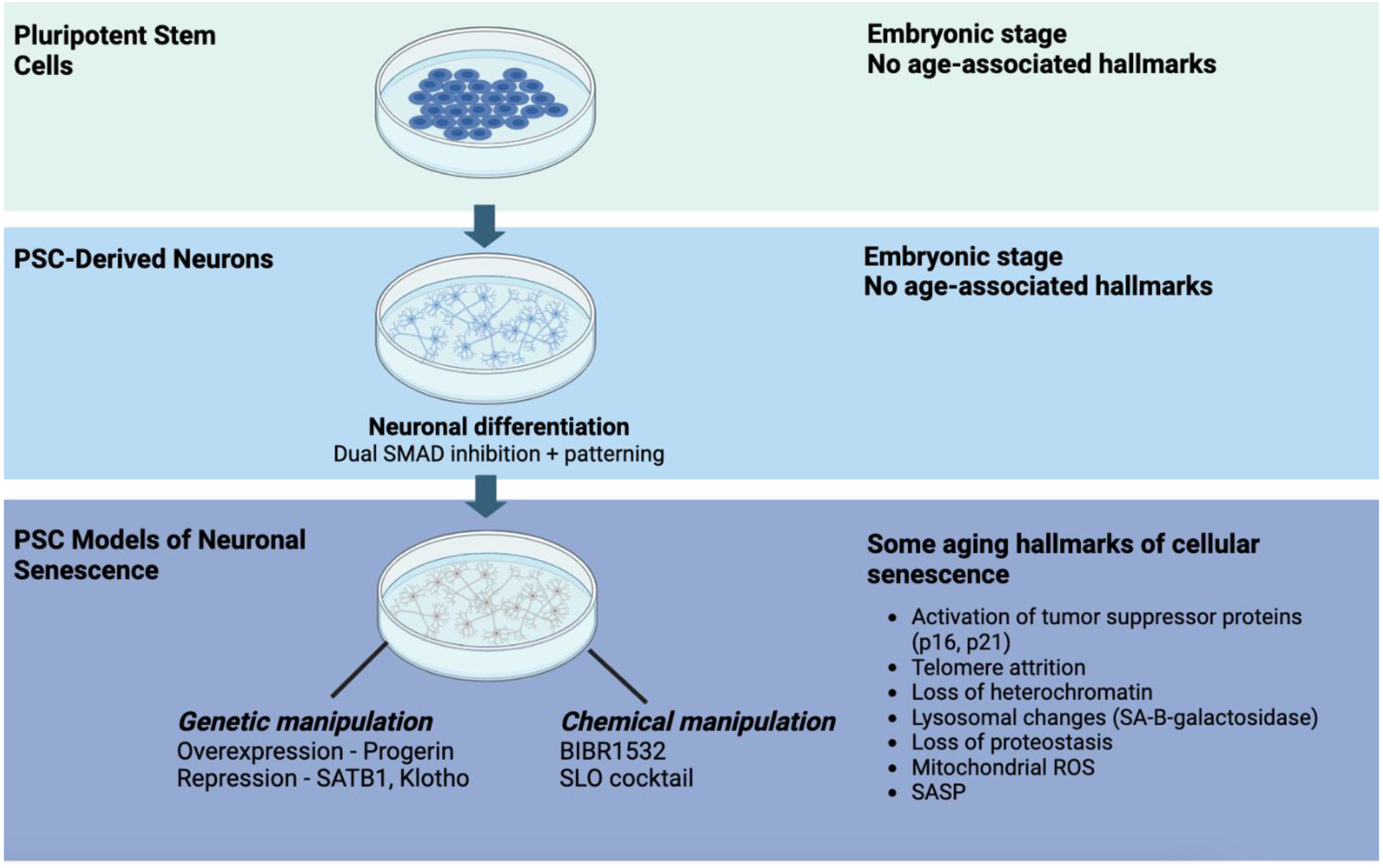
Summary of pluripotent stem cell models and associated aging phenotypes BIBR1532 is a selective human telomerase inhibitor. SLO cocktail is a combination of three drugs including SBI-0206965 (autophagy inhibitor), Lopinavir (HIV protease inhibitor) and O151 (DNA glycosylase-1 inhibitor).

*In vitro* models of human neurons provide a complimentary system to study cellular senescence. These are particularly valuable as they can be generated from specific patients and can be used to explore cellular and molecular pathological events that may represent early stages of neurological disease onset. One approach to model neuronal senescence is the direct conversion of fibroblasts or other aged somatic cell into induced neurons (iNs), which by-passes the pluripotent stage and thereby preserves several disease- and age-associated signatures^52–54^. Indeed, studies of adult individuals and of primary tauopathy patient-derived neurons have demonstrated that the isoform (4R) of Tau, particularly critical to disease, can be readily detected in iNs but is typically not observed in iPSC-derived neurons^55^. In addition, features of cellular senescence have been identified in iNs derived from both sporadic and familial AD patients^6^. Furthermore, the iNs showed sensitivity to senolytic treatment and the enhanced expression of the senescence marker p16 *in vitro* was correlated to p16 expression in a cohort of patient AD brains^6^. However, whether the full biological process of aging is conserved after iN reprogramming and to what extent the retained aging signatures reflect neuronal aging versus aging of the somatic donor cell remains to be fully determined. For example, a recent study suggests that iNs reprogrammed from fibroblasts retain both the donor fibroblast and neuronal aging signatures^56^. Furthermore, whilst progress has been made in direct reprogramming technologies, the approach still has challenges such as the relatively moderate yield of induced neurons, and difficulties in maintaining iNs for extended *in vitro* periods. One potential solution to improve yield may be the reprogramming of fibroblasts into iNs in a 3D environment^57^. An alternative approach to model neuronal senescence is the directed differentiation of human pluripotent stem cells (hPSC) into neurons. Since the inception of pluripotent stem cell (PSC) lines either derived from blastocyst stage embryos^58^ or via transcription factor-based reprogramming from embryonic/adult somatic cells^59^, there has been significant efforts to generate defined, high-purity neuronal cultures from hPSC. In fact, many robust protocols are available for generating a broad range of neuronal subtypes including cortical, dopaminergic or spinal motor neurons among others^60–63^. However, regardless of the donor age, induced PSCs (iPSCs) and their progeny resemble an embryonic age that is indistinguishable from that of their embryonic stem cell-based counterpart^64^. In essence, donor somatic cells undergo cellular rejuvenation by an erasure of their age-associated signatures, including markers of cellular senescence (Figure 2)^37,64–66^. Directed differentiation of hPSCs gives rise to embryonic-stage human neurons that progressively mature and age at a pace that follows a human specific clock of development^55^. For example, the transcriptional profile of directly reprogrammed neurons can be segregated from that of PSC-derived neurons by the activation of p16, p21 and several other age-associated markers^55^. As a consequence, various methods are being explored to introduce aging phenotypes (including senescence) into human PSC-derived neurons (see Table 2), to enable more rapid, effective and reliable modelling of late-onset neurodegenerative diseases. However, whether the senescence-induced neurons reflect the full biological process of aging, or if bona fide cellular senescence can be induced in PSC-derived neurons, will be discussed below.

## Methods to introduce hallmarks of cellular senescence in hPSC-derived neurons

Spontaneous emergence of senescent cells has been observed upon *in vitro* culture of hPSC-derived lineages. For example, prolonged culture of human cortical organoids has been reported to elicit cellular and metabolic stress which can impair cell-type specification^67^ but also lead to the development of senescence-related features^28^. Aged cortical organoids exhibit enhanced SA-β-gal lysosomal activity, as well as elevated expression of factors known to regulate cell cycle arrest (p21/p16) and SASP (IL-8, IL-1α, IL-1β) typically by 10 weeks^28^. Significant up-regulation of multiple senescence-associated genes (ELMOD1, SLC9A7, TAP1, CCND3, LRP10) across 3 independent scRNAseq datasets of cortical organoids have also been noted^28,68–70^. Similarly, cultured cortical neurons (>10weeks) display increased SA-β-gal and p16, with a notable reduction in telomere fluorescence intensity and neurite diameter^28^. However, in all those instances, it could be argued that prolonged culture leads to stress-induced expression of senescence markers rather than modelling physiological aging or senescence.

In recent years, there has been a significant advancements in the transplantation of hPSC-derived neuronal subtypes^61,62,71–74^ which may offer a more physiologically relevant model to study aging or senescence. One concern is the relatively immature age of the engrafted neurons (even at study endpoints), which could potentially be overcome by the generation and transplantation of iN-derived organoids that retain age-associated signatures, but this strategy has not yet been demonstrated.

An alternative approach to accelerate cellular age in hPSC-derived lineages is to manipulate genes associated with premature aging syndromes, such as Hutchinson-Gilford progeria syndrome. Forced expression of progerin has been suggested to trigger neuronal senescence in a mouse model of Huntington’s disease but results have been inconsistent across studies (discussed above). In hPSC-derived populations, over-expression of progerin has been shown to induce multiple age-related phenotypes in fibroblasts, mDA neurons and striatal neurons (Table 2)^37,47^. However, in contrast to fibroblasts, progerin treatment in iPSC-derived mDA and striatal neurons did not robustly upregulate the senescence marker SA-β-gal and heterochromatin markers (HP1γ, H3K9me3), and Lap2α were unchanged, demonstrating a distinct cell-type specific response to progerin^37^. However, progerin-induced aging-like phenotypes in iPSC-derived dopamine neurons resulted in some dopamine-specific age-related phenotypes including accumulation of neuromelanin and progressive loss of tyrosine hydroxylase expression in grafted neurons, as well as a pronounced dendrite degeneration phenotype *in vitro*^37^. These characteristics were exacerbated in dopamine neurons derived from two genetic PD iPSC models. While the progerin approach can model several aspects of neuronal aging, it does not capture the full range of age-related phenotypes. Furthermore, it requires ectopic expression of progerin in neurons given the low endogenous levels of Lamin A. Accordingly, progerin-based models may represent a pathological form of aging rather than simply accelerating the physiological aging process. Investigation into other premature aging candidates, such as ERCC1 (discussed above), that specifically cause accelerated senescence, may be particularly relevant, though the complete loss of ERCC1 triggers neuronal death rather than senescence^75^.

Another gene shown to be involved in the process of aging is the longevity gene Klotho. Klotho (KL) is readily detectable in many tissues of the body (including several brain cell types) and expression levels progressively decline with advancing age^76–78^. Expression of Klotho in the CNS is most prominent in the choroid plexus, with lower expression in Purkinje neurons of the cerebellum, cortical neurons, spinal motor neurons, hippocampal neurons, dopaminergic neurons and oligodendrocytes^78,79^. The relevance of Klotho in brain aging has been studied using klotho-deficient mice which rapidly develop cognitive deficits and exhibit selective loss of dopaminergic neurons^80^. Dopaminergic neuron loss is thought to be a result of increased oxidative stress^80^. In addition, KL deficient mice have significantly fewer Purkinje neurons in the cerebellum, decreased oligodendrocyte number and reduced anterior horn spinal MNs, and show a reduced lifespan^77^. Cellular senescence is yet to be reported in Klotho-deficient mice. A recent report has investigated the impact of perturbing Klotho expression in hPSC-derived cortical neurons. Interestingly, transcriptional repression of Klotho in cortical neurons accelerated culture-induced neuronal senescence via upregulation of p21 and p16^INK4A^ in both 2D neurons and organoids^28^. Conversely, up-regulation of this gene attenuated cellular senescence in cortical neurons.

Another approach to study neuronal senescence is to investigate genetic variants associated with neurodegenerative disease. SATB1, a master transcriptional regulator, has recently been identified as a risk factor for Parkinson’s disease (PD)^81,82^. Interestingly, post-mortem studies of PD brains have noted a reduction in SATB1 activity within brain regions associated with PD pathology^83^. Genetic deletion of SATB1 in human embryonic stem cell-derived DA neurons, but not cortical neurons, can induce a cellular senescence transcriptional program via a p21-dependent mechanism. These neurons display a host of common hallmarks that are central to cellular senescence (see Table 2) and show sensitivity to a diverse panel of senolytic compounds^32^. Importantly, the senescent phenotype can be mostly reversed by inhibition of p21 in SATB1^KO^ neurons^32^. The cell type specific response to loss of SATB1 raise the question whether subtypes of neurons are more vulnerable to senescence and which of the genes that regulate neuronal senescence are specific to a given neuronal subtype. It also remains to be explored if induction of cellular senescence by loss of SATB1 can accelerate the presentation of disease phenotypes in PD DA neurons.

Several pharmacological treatments that elicit senescence phenotypes have been explored in hPSC-derived neurons. The prolonged exposure of hPSCs to BIBR1532, an inhibitor of telomerase catalytic activity, during both the pluripotent stage prior to differentiation and maintained during dopamine neuron differentiation, result in neurons with an increased percentage of critically short telomeres. Those neurons derived from BIBR1532 treated cultures also show other age-related features, such as increased DNA damage, a reduced number and length of dendrites and increased mitochondrial ROS^38^. Several of these aging phenotypes are observed during cellular senescence but additional profiling of markers central to cellular senescence, such as SA-β-gal, p16/p21/p53 and SASP, will be required to address whether telomere attrition in hPSCs by inhibition of telomerase catalytic activity results in neurons that exhibit bona fide senescence. Interestingly, the same BIBR1532 treatment paradigm led to premature loss of TH expression *in vitro* similar to the findings following progerin overexpression in grafted dopamine neurons.

A recent drug screen on neonatal fibroblasts has identified a series of small molecules that can induce senescence features without causing significant DNA damage^35^. From these candidates, the authors identified that the combination of SBI-0206965 (autophagy inhibitor), Lopinavir (HIV protease inhibitor) and O151 (DNA glycosylase-1 inhibitor), named SLO, effectively enhanced neuronal senescence. Specifically, SLO-treated neurons elicited SA-β-gal with a loss of H3K9Me3, HP1γ and Lap2β expression. Further assessment revealed disrupted proteostasis with more extensive protein aggregation and mitochondrial defects, but no changes in Lamp2A. These phenotypes were validated in both cortical and motor neurons. SLO-treated cortical neurons had a high overlap of age-related transcripts with iNs derived from aged fibroblasts and with aged cortex, as well as up-regulation of genes involved in Hutchinson-Gilford Progeria syndrome. They further tested the influence of senescence on disease-related phenotypes in motor neurons (MNs) from TARDP mutant ALS and isogenic iPSCs. SLO treatment accelerated ALS-specific neurodegeneration in TARDP MNs, most strikingly by inducing increased levels of phosphorylation of TDP43, but also protein aggregation and axonal degeneration^35^.

Another means to induce aging is to manipulate the epigenetic status of the cell. As we age, the epigenetic landscape changes in the human body as a consequence of dsDNA breaks, which causes chromatin modifying proteins to relocate to the site of these breaks as a part of the DNA repair machinery^5^. This results in a loss of epigenetic information which appears to accelerate aging. A novel reversible method to manipulate this process, defined as ICE (inducible changes to the epigenome), has recently been investigated in a mouse model to accelerate hallmarks of aging, including senescence, which leads to an advancement of the DNA methylation clock among other phenotypes^84^. While this approach has yet to be applied for hPSC-derived neurons, it is an exciting avenue for further investigation.

Of interest, a recently established transcriptional method to score cellular aging (RNAge) has confirmed that several of the paradigms (SATB1 loss, progerin over-expression and chemical induction of senescence – SLO) discussed in the review increased cellular age in hPSC-derived neurons^56^.

While the primary focus of this review is neuronal senescence in aging and neurodegenerative disease, there are other cell types of the CNS that can be derived from iPSCs and have been shown to express features of cellular senescence in aged and neurodegenerative states^23,85,86^. However, distinguishing senescence-associated glia from other primed and reactive inflammatory states remains a challenge for the field, as reviewed by Ng et al.,^7^. Nonetheless, it will be important to determine which of these senescent cell types are drivers of neurodegenerative disease and which play the most critical role in neurodegeneration.

## Conclusion

Each of the strategies to trigger age-related markers or senescence in hPSC-derived neurons caused a unique subset of hallmarks that are associated with cellular senescence (Figure 2). However, the heterogeneity in cellular phenotypes is not surprising given the high phenotypic diversity present in other senescent cell types and given the distinct methods to induce and measure senescence^16,87,88^. Furthermore, it also seems likely that neuronal subtype contributes to whether a given senescence marker is induced or not. These findings highlight the complexity and intricacies of cellular senescence, and it remains to be determined if any of these approaches can reproducibly model the full biological process of senescence in a manner that reflects age-related senescence in human neurons. It is possible that each inducer only mimics aspects of the process and that a combination of methods will be required to induce a more complete set of neuronal senescence markers. All of those efforts in hPSC-derived neurons will complement the use of directly reprogrammed iNs that retain age-related senescence features but present challenges in recreating the full scale and diversity of neuronal and glial cell types available using hPSC-based approaches.

**Figure 3:**
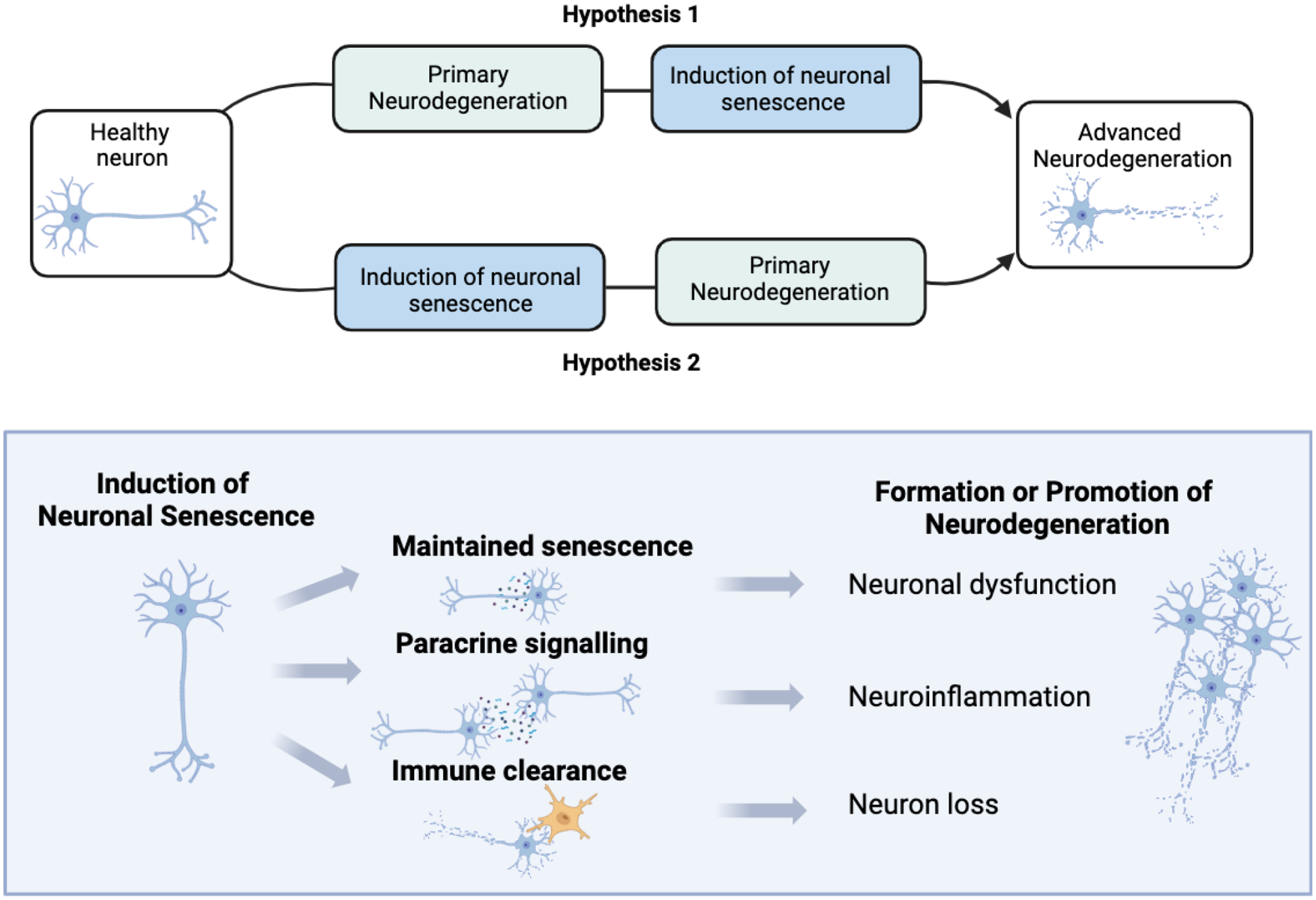
Hypothetical mechanisms of neuronal senescence that could be implicated in neurodegenerative disease The top part of the schematic outlines two hypotheses of disease progression in neurons in relation to neuronal senescence. The remainder of the schematic proposes biological mechanisms associated with neuronal senescence and suggests how this could impact disease onset and/or progression.

More broadly, the relatively immature state of neuronal subtypes derived from hPSC is now widely recognized as a challenge for the study of age-related diseases. We have discussed some of the preliminary efforts to induce neuronal senescence in patient-derived iPSCs which can advance the appearance of some disease-related phenotypes in PD and ALS^35,37,38^. However, this field is still at its infancy and many open question remain such as: is cellular senescence a cause or consequence of neurodegenerative disease and how does it relate to disease progression, can senescence spread from neuron to neuron or across other cell types in the CNS and how does senescence impact neuronal function and age-related synaptic changes (see Figure 3 for hypothetical mechanisms of how neuronal senescence could be implicated in disease). Finally, it will be important to determine whether senescence is reversible, and to assess the therapeutic potential of senolytic drugs in modulating neuronal senescence, and if the elimination of senescent cells, including senescent neurons, will be the most beneficial approach.

In this review, we have highlighted a panel of senescence-related markers that can be used to identify senescence neurons. However, we recommend that future studies will have to standardize those readouts and to systematically compare the same readouts across the many induced aging paradigms. Furthermore, it will be important to move beyond this framework and to include analyses of neuronal function that may arise in response to cellular senescence – such as modifications to neuronal activity, the status of synaptic protein(s) and potentially adverse cell to cell interactions via SASP. Finally, it will be critical to apply strategies for controlled manipulation of the epigenetic landscape in altering senescence induction and the functional consequences thereof.

## Future directions

Cellular senescence in neurodegenerative disease is an emerging field, but there are still many challenges that hamper progress. Lacking in the field is a clear definition of senescence at the cellular, molecular, transcriptional and epigenetic level in both the mouse and human brain. Efforts to achieve this goal at single cell resolution are currently underway, particularly to identify conserved or unique senescence signatures across cell types and identify biomarkers with improved specificity across human age. Several initiatives have been established to address the challenges associated with cellular senescence including the NIH SenNet consortium^89^. Transcriptomic/proteomic atlases are also being developed to stratify senescence genes across multiple healthy human tissues and for various senescence inducers^16,87,88^. One of these reports identified three novel SASP genes (GDF15, STC1, SERPINS) which significantly correlate with age of human plasma and overlap with several aging markers^90^. Expansion of these atlases to encompass neurons and various neuronal subtypes (and other known senescent CNS cell types) and to cross-compare those data with data from iPSC-derived neurons are needed.

Epigenetic clocks have been developed as a tool to predict biological age based on changes in DNA methylation from embryogenesis to old age. These clocks can be applied across multiple tissues, including the brain^91–93^ and tailored to capture DNA-methylation based, age-related changes in iPSC and iPSC-derived neurons^94^. The recent development of a universal mammalian clock from the Horvath group identified clock loci associated with genes linked to cellular senescence, such as ERCC1 and BDNF^95–97^, presenting a novel tool to study the link of age- and senescence-related changes.

Of relevance to modelling neuronal senescence *in vitro*, it will be important to assess if the senescent profile of neurons from human brain tissues are comparable to *in vitro*, patient-derived iPSC neurons. A convergence of approaches and methods will be required to eventually disentangle phenotypic diversity of senescent states that arise depending on the neuronal cell types studied and the strategies used to trigger senescence-like states in hPSC-derived lineages. Such efforts will be critical to harness the full potential of those approaches in modelling late onset neurological disorder.

## Figures and Tables

**Table 1:** Summarisation of senescent phenotypes detected in primary cultured and aged rodent neurons and human PSC-derived neurons

Neuronal senescence phenotypes	Markers	Assays/Models	Publications
**Activation of tumour suppressor pathways**	p16/RB	Fluorescent reporter mouse models (p16-3MR, p16-cre, p16-Tdtom+, p16-luciferase)	^27–29^
	p21/p53	Fluorescent reporter mouse models (p21-Cre)	^28,30–32^
**Lysosomal changes**	β-galactosidase activity at pH 6.0	SA-β-gal assay, Cell event Senescence	^28,30–36^
	Lipofuscin	Sudan Black B	^31^
	Lysosomal content	LysoTracker	^32^
**DNA damage response**	γ-H2AX	IHC/high content imaging, flow-based assays	^28,32,37,38^
**Nuclear changes**	LaminA/C, LAP2β, LAMP1/2, LamB1, Nucleus size	IHC/high content imaging, flow-based assays	^28,32,35,37,38^
**Chromatin modifications**	HP1γ, H3K9Me3	IHC/high content imaging, flow-based assays	^35^
**Loss of proteostasis**	Proteostat	ProteoStat aggresome detection kit	^35^
**Mitochondrial changes**	Mitochondrial ROS Mitochondrial membrane potential Mitochondrial morphology	MitoSox JC-10 Mitotracker	^35,37,38^
**Telomere alterations**	Telomerase activity Telomere length	TRAP assay HT-QFISH	^38^
**SASP**	IL-6, MCP-1, IGFBP7, IL-8, IL-1α, IL-1β	Multiplex assay, ELISA	^30–32^

Reactive oxygen species (ROS) Telomerase Repeated Amplification Protocol (TRAP), High-throughput quantitative fluorescence in situ hybridization (HT Q-FISH).

**Table 2.** Overview of *in vitro* human pluripotent stem cell models to study features of cellular senescence in neurons

Neuronal subtype	Disease-related mutations	Method to induce cellular senescence	Phenotypes	Markers
hPSC-derived cortical neurons^28^	None	Extended culture	Lysosomal changes	**↑**SA-β-gal
			Activation of tumour suppressor pathways	**↑**p21, **↑**p16
			SASP	IL-8, IL-1α, IL-1β
hPSC-derived dopamine neurons^35,38^	Healthy and PD PINK1/PARKIN mutants	Inhibitor of telomerase activity (BIBR1532)	DNA damage	**↑** γ-H2AX
			Mitochondrial changes	**↑**Mitochondrial ROS
			Telomere attrition	**↑** Telomerase activity, **↑**% short telomeres
hPSC-derived cortical and motor neurons^35^	Healthy and ALS TDP-43 mutants	Senescence cocktail (SLO: SBI-0206965, Lopinavir, O151)	Lysosomal changes	**↑** SA-β-gal
			Nuclear changes	**↓**Lap2β, No change in LAMP2
			Chromatin modifications	**↓**H3K9Me3, **↓**HP1γ
			Loss of proteostasis	**↑**Proteostat
			Mitochondrial changes	**↑**Mitochondrial ROS, **↓**MMP, **↓**Mitochondrial area and branch length
hPSC-derived dopamine neurons^37^	Healthy and PD PINK1/PARKIN mutants	Progerin overexpression	Lysosomal changes	No changes in SA-β-gal
			DNA damage	**↑**γ-H2AX
			Nuclear changes	No change in Lap2α
			Chromatin modifications	No change in HP1γ
			Mitochondrial changes	**↑**Mitochondrial ROS
hPSC-derived striatal neurons^47^	Healthy and HD CAG repeat mutants	Progerin overexpression	DNA damage	**↑**γ-H2AX
			Neurite changes	Loss of dendritic complexity and length
hPSC-derived dopamine neurons^32^	None	Deletion of SATB1	Lysosomal changes	**↑**SA-β-gal, **↑**LysoTracker content
			Activation of tumour suppressor pathways	**↑**p21, No change p16^INK4A^/p53
			Nuclear changes	**↓**Lamβ1, **↓**LAMP1, enlarged nucleus
			SASP	Multiple interleukins, chemokines, growth factors
hPSC-derived cortical neurons^28^	None	Klotho repression	Activation of tumour suppressor pathways	**↑**p21, **↑**p16^INK4A^
